# Origin, evolution, and diversification of inositol 1,4,5-trisphosphate 3-kinases in plants and animals

**DOI:** 10.1186/s12864-024-10257-7

**Published:** 2024-04-08

**Authors:** Tao Xiong, Zaibao Zhang, Tianyu Fan, Fan Ye, Ziyi Ye

**Affiliations:** 1 School of Life and Health Science, Huzhou College, Huzhou, Zhejiang China; 2 College of Life Science, Xinyang Normal University, Xinyang, Henan China; 3 College of Forestry and Biotechnology, Zhejiang A & F University, Hangzhou, Zhejiang China

**Keywords:** IP3K family, Gene structure, Expression pattern, Evolutionary history, Gene duplication

## Abstract

**Background:**

In Eukaryotes, inositol polyphosphates (InsPs) represent a large family of secondary messengers and play crucial roes in various cellular processes. InsPs are synthesized through a series of pohophorylation reactions catalyzed by various InsP kinases in a sequential manner. Inositol 1,4,5-trisphosphate 3-kinase (IP3 3-kinase/IP3K), one member of InsP kinase, plays important regulation roles in InsPs metabolism by specifically phosphorylating inositol 1,4,5-trisphosphate (IP3) to inositol 1,3,4,5-tetrakisphosphate (IP4) in animal cells. IP3Ks were widespread in fungi, plants and animals. However, its evolutionary history and patterns have not been examined systematically.

**Results:**

A total of 104 and 31 IP3K orthologues were identified across 57 plant genomes and 13 animal genomes, respectively. Phylogenetic analyses indicate that IP3K originated in the common ancestor before the divergence of fungi, plants and animals. In most plants and animals, IP3K maintained low-copy numbers suggesting functional conservation during plant and animal evolution. In Brassicaceae and vertebrate, IP3K underwent one and two duplication events, respectively, resulting in multiple gene copies. Whole-genome duplication (WGD) was the main mechanism for IP3K duplications, and the IP3K duplicates have experienced functional divergence. Finally, a hypothetical evolutionary model for the IP3K proteins is proposed based on phylogenetic theory.

**Conclusion:**

Our study reveals the evolutionary history of IP3K proteins and guides the future functions of animal, plant, and fungal IP3K proteins.

**Supplementary Information:**

The online version contains supplementary material available at 10.1186/s12864-024-10257-7.

## Introduction

Inositol polyphosphates (InsPs) are a class of signaling molecules that play vital roles in various cellular functions, such as apoptosis, mRNA export, DNA repair, embryogenesis, stress response, membrane trafficking and gene expression [1–4]. Inositol 1,4,5-trisphosphate (InsP3) is the best characterised InsP that acts as a second messenger in mediating Ca^2+^ release from the endoplasmic reticulum [5–8]. The cellular synthesis of InsPs are catalyzed by different InsP kinases, with inositiol polyphosphate kinase (IPK) represents the most characterised one [9]. The IPK superfamily consists of three distinct subgroups, the inositol 1,4,5-trisphosphate 3-kinase (IP3K), the inositol phosphate multikinases (IPMK, Arg82 or Ipk2) and inositol hexakisphosphate kinases (IP6K, Kcs1) [9–11]. These three kinase subgroups display significant defferences in substrate specificity, distribution, expression, regulation and function [9].

Mammalian and Human IP3Ks catalyze a single reaction that specifically phosphorylate inositol 1,4,5-trisphosphate (IP_3_) to inositol 1,3,4,5-tetrakisphosphate (IP_4_) [12–15]. The mammalian IP6Ks and yeast Kcs1 phosphorylate the C5 position of inositol hexakisphosphate (IP_6_) and 1-InsP_7_ to generate 5-InsP_7_ and 1,5-InsP_8_, respectively [1, 16–18]. Plants lack the canonical IP6K-type proteins [19]. However, two *Arabidopsis thaliana* ITPK1 and ITPK2 were reported to phosphorylate IP_6_ to generate 5-InsP_7_ in vitro and in vivo [20–22]. Human IPMKs can catalyze more substrates and possess 6-kinase activity toward Ins(1-5)P_4_, 3-kinase activity toward IP_3_ and Ins(1, 4-6)P_4_, 5-kinase activity toward Ins(1,3,4,6)P_4_, and phosphatidylinositol 4,5-bisphosphate (PtdIns(4,5)P_2_) 3-kinase activities that phosphorylate PtdIns(4, 5)P_2_ to PtdIns(3,4,5)P_3_[23]. Yeast and plant IP3Ks, also known as inositol polyphosphate kinase (IPK) and inositol phosphate multikinase (IPMK), also display a broad catalytic activity towards multiple inositol phosphates [9, 24]. Yeast IPK2/IP3K (also called Arg82/ArgRIII) is a dual-specificity IP_3_/IP_4_ 6/3-kinase that sequentially phosphorylates IP_3_ to 1,4,5,6-tetrakisphosphate (IP_4_) to 1,3,4,5,6-pentakisphosphate (IP_5_) in vivo [17, 25, 26]. Also, yeast IP3K has a 5-kinase activity toward 1,3,4,6-tetrakisphosphate (IP_4_) and 1,2,3,4,6-pentakisphosphate (IP_5_) and a 5P-kinase activity toward 1,3,4,5,6-pentakisphosphate (IP_5_) in vitro [27, 28]*.* Similar to yeast IPK2, *A. thaliana* IP3Ks (IPK2, IPMK) are also a dual-specificity 6/3-kinase that phosphorylate IP_3_ to IP_4_ to IP_5_ in vivo [17, 25, 29]. In addition, *A. thaliana* IP3K also display a 5-kinase activity to phosphate 1,3,4,6-tetrakisphosphate (IP_4_) and 1,2,3,4,6-pentakisphosphate (IP_5_) to generate 1,3,4,5,6-pentakisphosphate (IP_5_) and IP_6_ in vitro*,* respectively [30, 31]. Recently, both in vitro and in vivo experiments demonstrate that one isoform of *A. thaliana* IP3K (AtIPK2α) can phosphorylate InsP_6_ to generate 4/6-InsP_7_ [32]. Therefore, IP3K, IP6K and IPMK have different substrate specificity, and mammalian and human IP3Ks and IPMK have (Ins(1,4,5)P_3_) 3-kinase activity, while yeast and plant IP3Ks are predominantly ins(1,4,5)P_3_ 6-kinases.

IP3K are involved in various biological processes in yeast, animals and plants. Three IP3K isoforms (A, B, and C) were identified in humans and rats, respectively [33–40]. Human and rat IP3K isoforms are different in molecular masses, intracellular distribution, tissue expression, and physiological functions. For example, rat IP3K-A is involved in F-actin binding and specifically expressed in brain and testes [41–43], IP3K-B is localized to ER and predominantly expressed in lung [44–46], while IP3K-C shuttles between cytoplasm and the nucleus, and is mainly present in heart, brain, and testis [39, 47]. These specific distribution and expression patterns contribute to their different physiological functions. IP3K-A functions in learning and memory via activity-dependent Rac scaffolding mechanism [48], IP3K-B functions in immune responses [49, 50], and the function of IP3K-C is still not clear. *A. thaliana* has two IP3K isoforms (AtIPK2α and AtIPK2β), with *AtIPK2α* functions in pollen germination and root growth [51], while *AtIPK2β* functions in axillary shoot branching, flowering, seed growth and seedling development [52–54]. Rice has one *IP3K* gene (*OsIPK2*), functions as an inositol polyphosphate multikinases, and plays a role in maintaining phosphate balance, promoting root development, and regulating leaf senescence [55–57]. Maize IP3K (IPK2, IPK) is exprssed in embryo and mutation of *ZmIP3K* reduced seed phytic acid content, indicating that *ZmIP3K* is responsibel for IP6 biosynthesis in developing seeds [58]. Yeast IPK2/IP3K localizes in nuclear and function in arginine metabolism [25, 59].

The amino acid sequence identity among IP3K, IPMK and IP6K is low [60]. However, these three kinase subgroups share several strictly conserved signature motifs and display a similar backbone fold [9, 23, 60–63]. The ATP binding site and the consensus sequence PxxxDxKxG for substrate binding is similar in IP3K, IPMK and IP6K [64]. However, the inositol binding domian (IP domain) display significantly divegence in both sequence and structure. In human IP3K, the IP domain consists of a five α-helices, rich in basic residues and spans a region of 60 residues, while in IP6Ks, IMPKs and yeast and plant IP3Ks, the IP domains are much shorter, lack three α-helices and spanning about 30 residues [9, 23, 60–63]. These structure differences explained the substrate specificity among IP3Ks, IPMKs and IP6Ks. In addition, animal IP3Ks have a conserved Ca^2+^/Calmodulin (CAM) binding domain in the N-terminal [65], while *A. thaliana*, yeast, and nematode IP3Ks lacks a consensus CaM-binding site [14, 66]. Therefore, animal IP3Ks are activated by CaM in a Ca^2+^-dependent manner, while *A. thaliana*, yeast, and nematode IP3Ks are insensitive to Ca2 + /CaM. An actin-binding domain was identified in the N-terminus of rat IP3K-A, which is responsible for F-actin binding [67]. An ER localization signal and a nuclear export signal (NES) has been identified at the N-terminus of rat IP3K-B and IP3K-C [39, 68], respectively, which were responsible for its localization. Yeast and *A. thaliana* IP3Ks are nuclear localized [25, 29], whereas no obvious nuclear localization signal (NLS) was identified in their sequence.

Although the functions of IP3Ks are gradually being elucidated, research on them is still limited to a few model species, such as human, rat, *A. thaliana*, rice, and yeast. At present, not much is known about its origin and evolution. A previous analysis indicated that IP3Ks, IP6Ks and IPMKs evolved from a common ancestor before the divergence of yeast, plants and animals, and IP6Ks emerged initially, followed by IPMKs and finally by IP3Ks [1, 10]. However, these phylogenetic classification relies on a few species, which limited a clear understanding of the evolutionary origin and phylogenetic relationships of IP3Ks. Therefore, a more accurate and complete phylogenetic system is needed to further classify the IP3Ks.

Here, we traced the evolutionary history of *IP3Ks* by searching the complete genome sequences of plants and animals. Our study provides a comprehensive perspective on the evolution of IP3Ks, explores their origins, evolutionary processes, and functional diversity, and provides a solid foundation for further functional resolution and molecular evolutionary studies.

## Results

### Identification and distribution of IP3K genes

IP3K protein sequnces in the genomes of 13 animals, 57 plants, and 3 fungi were identified with the Hidden Markov Modeling algorithm and BLASTP search (Table 1). The retrieved proteins were examined by SMART, PFAM, and SWISS-MODEL, and candidates containing IPK domain and displaying an identical 3D structures to yeast, human and *A. thaliana* IP3Ks were recognized as "true" IP3K proteins and used for subsequent analysis. The copy number of IP3K protein varies in different animal and plant lineages. In early invertebrates, such as *C. elegans*, *N. vectensis*, and *C. intestinalis*, IP3K is a single copy, while in vertebrates its copy was expanded with 4, 3, and 3 IP3K isoforms were identified in zebrafish, human and rat, respectively. In plants, *IP3K* genes are present in major lineages of green plants, including algae, bryophyta, gymnosperms and angiosperms. In most plant lineages (Chlorophyta, Bryophyta, Pteridophyta, Gymnosperm, and Monocots), the copy numbers are nearly constant (e.g. only one copy is found for most plants). While in most dicots, *IP3K* is expanded with more copy numbers were identified. In fungi, the copy number of IP3K is constant with two copies were identified in each species (Table 1). The copy number of IP3K in animals and plants has changed during evolution, and the increase in copy number may be related to the increase in biological complexity.

**Table 1:** The number of IP3K proteins in animals, plants, and fungi

Taxonomy			Number of Species	Species name	Abbr	Number of IP3K	Average number of IP3K per species
Animals	Invertebrates		5	*Caenorhabditis elegans*	Ce	1	1.40
				*Nematostella vectensis*	Nv	1	
				*Ciona intestinalis*	Ci	1	
				*Drosophila melanogaster*	Dm	2	
				*Branchiostoma floridae*	Bf	2	
	Vertebrates		8	*Homo sapiens*	Hs	3	3.00
				*Pan troglodytes*	Pt	3	
				*Mus musculus*	Mm	3	
				*Danio rerio*	Dr	4	
				*Bufo gargarizans*	Bg	3	
				*Takifugu rubripes*	Tr	3	
				*Mauremys reevesii*	Mr	3	
				*Gallus gallus*	Gg	2	
Plants	Chlorophyta		3	*Chlamydomonas reinhardtii*	Cr	1	1.00
				*Volvox carteri*	Vc	1	
				*Ostreococcus lucimarinus*	Ol	1	
	Bryophyta		1	*Physcomitrium patens*	Pp	1	1.00
	Pteridophyta		1	*Selaginella moellendorffii*	Sm	2	2.00
	Gymnosperm		1	*Gnetum montanum*	Gn	1	1.00
	Basal angiosperms		1	*Amborella trichopoda*	Atr	1	1.00
	Angiosperms	Monocots	9	*Oryza sativa*	Os	1	1.33
				*Zea mays*	Zm	3	
				*Sorghum bicolor*	Sb	1	
				*Setaria italica*	Sit	1	
				*Panicum virgatum*	Pvi	2	
				*Brachypodium distachyon*	Bd	1	
				*Musa acuminata*	Ma	1	
				*Dendrobium catenatum*	Dec	1	
				*Dioscorea cayenensis*	Dic	1	
		Dicots	41	*Aethionema arabicum*	Aa	2	2.05
				*Arabidopsis halleri*	Ah	2	
				*Arabidopsis lyrata*	Al	2	
				*Arabidopsis thaliana*	At	2	
				*Arabis alpina*	Aal	2	
				*Barbarea vulgaris*	Bv	2	
				*Boechera retrofracta*	Br	2	
				*Boechera stricta*	Bs	2	
				*Brassica napus*	Bn	6	
				*Brassica oleracea*	Bo	3	
				*Brassica rapa*	Bra	3	
				*Capsella grandiflora*	Cg	2	
				*Capsella rubella*	Cru	2	
				*Cardamine hirsuta*	Ch	2	
				*Eutrema salsugineum*	Es	2	
				*Isatis indigotica*	Ii	2	
				*Raphanus sativus*	Rs	5	
				*Schrenkiella parvula*	Sp	3	
				*Sisymbrium irio*	Si	2	
				*Thlaspi arvense*	Ta	2	
				*Carica papaya*	Cp	1	
				*Glycine max*	Gm	1	
				*Medicago truncatula*	Mt	2	
				*Phaseolus vulgaris*	Pv	1	
				*Aquilegia coerulea*	Ac	3	
				*Citrus sinensis*	Cs	1	
				*Citrus clementina*	Cc	1	
				*Cucumis sativus*	Csa	1	
				*Eucalyptus grandis*	Eg	2	
				*Fragaria vesca*	Fv	1	
				*Malus domestica*	Md	3	
				*Prunus persica*	Ppe	1	
				*Manihot esculenta*	Me	2	
				*Ricinus communis*	Rc	1	
				*Gossypium raimondii*	Gr	3	
				*Theobroma cacao*	Tc	1	
				*Populus trichocarpa*	Ptr	2	
				*Vitis vinifera*	Vv	1	
				*Solanum tuberosum*	St	2	
				*Solanum lycopersicum*	Sl	2	
				*Lactuca sativa*	Ls	2	
Fungi			3	*Saccharomyces cerevisiae*	Sc	2	2.00
				*Schizophyllum commune*	Sco	2	
				*Shizpsaccharomyces pombe*	Spo	2	

**Fig. 1:**
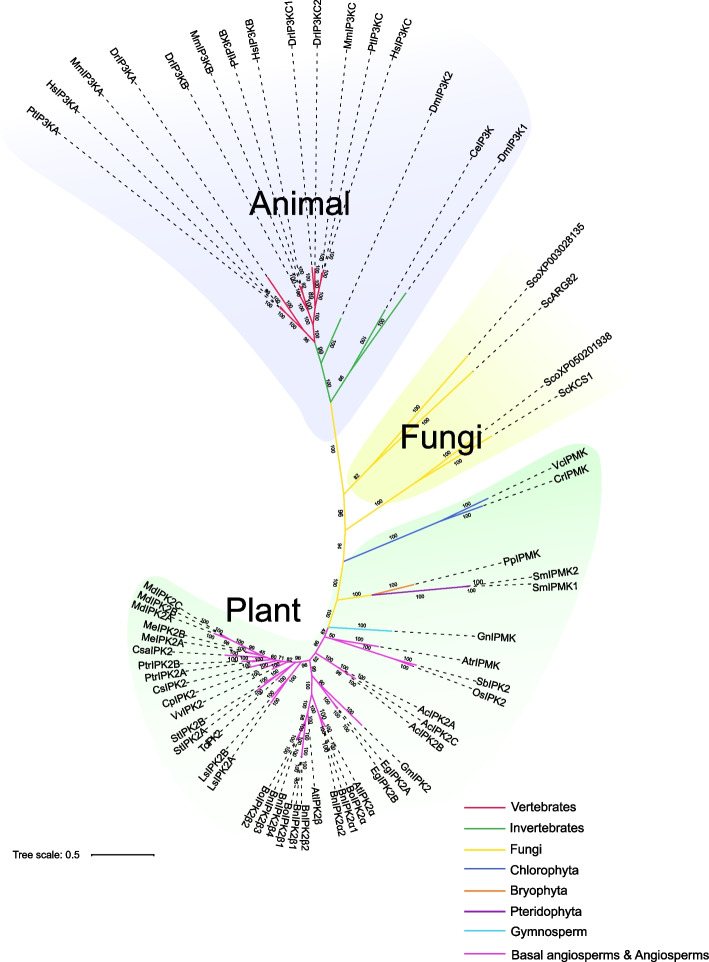
Phylogenetic relationship of representative *IP3K* genes from plants, animals and fungi. Phylogenetic tree was constructed using the Bayesian method. The IP3Ks derived from different lineages are shown in different colors. Pt: *P. troglodytes*, Hs: *H. sapiens*, Mm: *M. musculus*, Dr: *D. rerio*, Dm: *D. melanogaster*, Ce: *C. elegans*, Sco: *S. commune*, Sc: *S. cerevisiae*, Vc: *V. carteri*, Cr: *C. reinhardtii*, Pp: *P. patens*, Sm: *S. moellendorffii*, Gn: *G. montanum*, Atr: *A. trichopoda*, Sb: *S. bicolor*, Os: *O. sativa*, Ac: *A. coerulea*, Gm: *G. max*, Eg: *E. grandis*, At: *A. thaliana*, Bo: *B. oleracea*, Bn: *B. napus*, Ls: *L. sativa*, Tc: *T. cacao*, St: *S. tuberosum*, Vv: *V. vinifera*, Cp: *C. papaya*, Cs: *C. sinensis*, Ptr: *P. trichocarpa*, Csa: *C. sativus*, Me: *M. esculenta*, Md: *M. domestica*

An unrooted phylogenetic tree was constructed based on the IP3K proteins of representative plant, animal and fungi (Fig. 1). The topology of the phylogenetic tree clearly separated plants, animals, and fungi IP3K into 3 distinct clades, indicating that *IP3K* had originated before the split of plants, animals, and fungi. In addition, the phylogenetic tree suggests that the divergence of plants and animals IP3Ks occurred after the emergence of plants and animals, respectively.

The physical and chemical characteristics of the identified IP3K were examined (Table S1). In fungi IP3Ks, the proteins range in size from 268 to 1197 amino acids, with molecular weights varies from 30,418.03 to 133,164.3 Da, and isoelectric points ranging from 4.56 to 9.47. In plant IP3Ks, the range in protein length from 223 to 367 amino acids, with molecular weights ranging from 24,364.8 to 40,926.79 Da, and isoelectric points ranging from 5.23 to 8.44. In animals, the IP3K proteins have a length range of 364–946 amino acids, with a molecular weight range of 41,477.59–102,673.4 Da, and an isoelectric point range of 5.01–9.58.

### Phylogenetic classification of plant IP3Ks

**Fig. 2:**
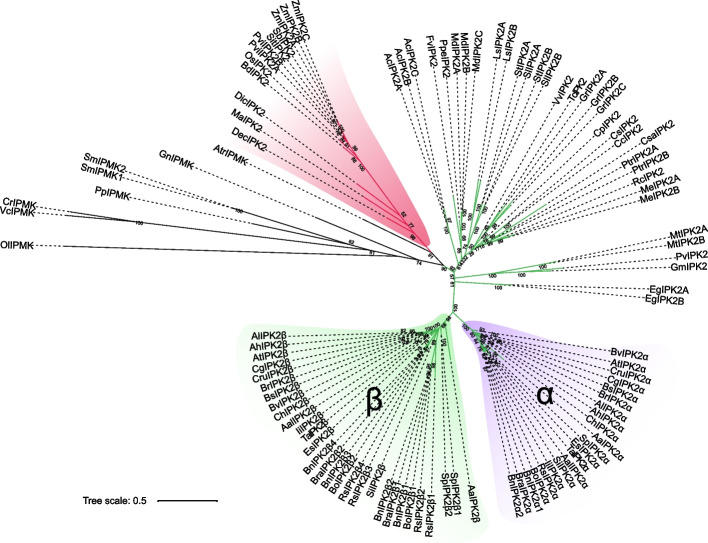
Phylogenetic relationship of *IP3K* genes in plants. Monocots are shown with red branching colors, and dicots are shown with green branching colors. Ol: *O. lucimarinus*, Vc: *V. carteri*, Cr: *C. reinhardtii*, Pp: *P. patens*, Sm: *S. moellendorffii*, Gn: *G. montanum*, Atr: *A. trichopoda*, Os: *O. sativa*, Zm: *Z. mays*, Sb: *S. bicolor*, Sit: *S. italica*, Pvi: *P. virgatum*, Bd: *B. distachyon*, Ma: *M. acuminata*, Dec: *D. catenatum*, Dic: *D. cayenensis*, Aa: *A. arabicum*, Ah: *A. halleri*, Al: *A. lyrata*, At: *A. thaliana*, Aal: *A. alpina*, Bv: *B. vulgaris*, Br: *B. retrofracta*, Bs: *B. stricta*, Bn: *B. napus*, Bo: *B. oleracea*, Bra: *B. rapa*, Cg: *C. grandiflora*, Cru: *C. rubella*, Ch: *C. hirsuta*, Es: *E. salsugineum*, Ii: *I. indigotica*, Rs: *R. sativus*, Sp: *S. parvula*, Si: *S. irio*, Ta: *T. arvense*, Cp: *C. papaya*, Gm: *G. max*, Mt: *M. truncatula*, Pv: *P. vulgaris*, Ac: *A. coerulea*, Cs: *C. sinensis*, Cc: *C. clementina*, Csa: *C. sativus*, Eg: *E. grandis*, Fv: *F. vesca*, Md: *M. domestica*, Ppe: *P. persica*, Me: *M. esculenta*, Rc: *R. communis*, Gr: *G. raimondii*, Tc: *T. cacao*, Ptr: *P. trichocarpa*, Vv: *V. vinifera*, St: *S. tuberosum*, Sl: *S. lycopersicum*, Ls: *L. sativa*

An IQ tree was constructed for IP3K proteins from green plant lineages including chlorophyta, bryophyta, pteridophyta, gymnosperm, basal angiosperms, and angiosperms (Fig. 2). The phylogenetic tree shows that the evolution of IP3K in plants coincides with the evolutionary relationship of species, clustered in a branch by species. In Brassicaceae, one gene duplication was occurred which gives the differentiation between IPK2α and IPK2β (Fig. 2). In addition, many lineage-spcific duplication events are identified, for 2, 3, 3, 3, 3, 2, 2, 2, 2, 2, 2, 2, 2, and 2 IP3K copies were identified in *S. moellendorffii*, *A. coerulea*, *M. domestic*, *G. raimondii*, maize, *Panicum virgatum*, *S. moellendorffii*, *M. truncatula*, *E. grandis*, *M. esculenta*, *P. trichocarpa*, *S. tuberosum*, *S. lycopersicum* and *L. sativa*, respectively. In Brassicaceae, most plants only have one gene corresponding to AtIPK2α and AtIPK2β, respectively (Fig. 2). In IPK2α branch, one gene duplication was occurred in *B. napus*. In IPK2β branch, one gene duplication was occurred in *B. oleracea*, *B. rapa*, *S. parvula*, and two gene duplication was occurred in *B. napus* and *R. sativus*, respectively (Fig. 2).

**Fig. 3:**
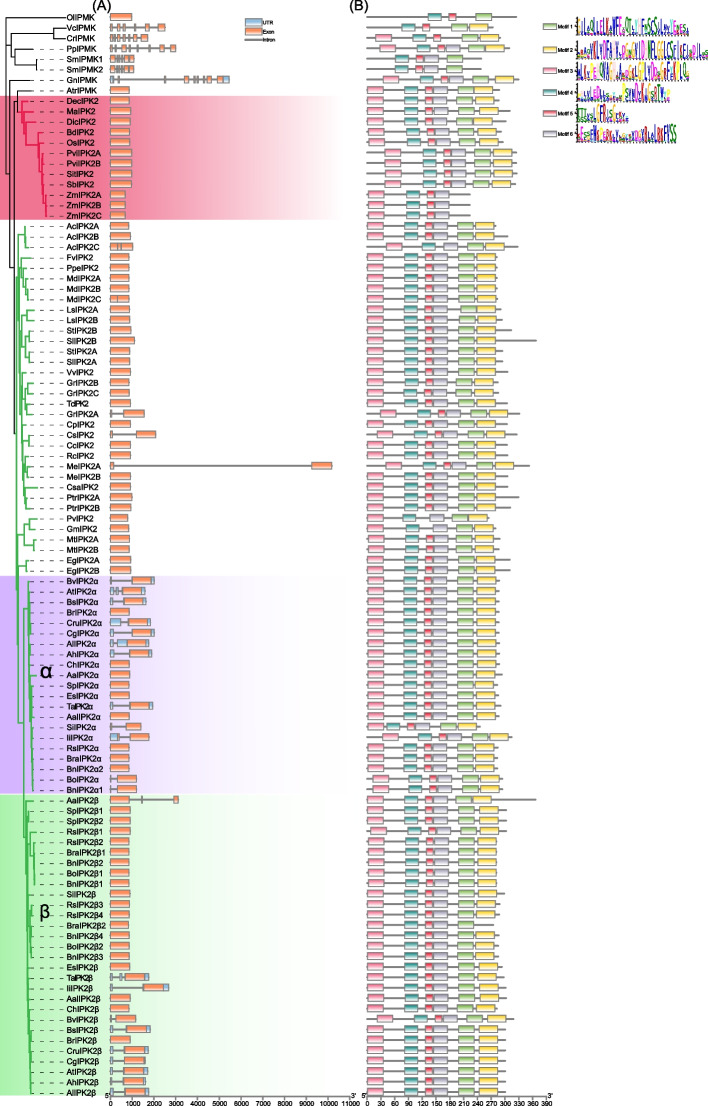
**A** Distribution of conserved motifs identified in proteins encoded by plants IP3K. **B** Gene structures showing the organization of exons and introns, plants *IP3K* genes. Monocotyledonous plants are shown with red background, and the α and β branches of the Brassicaceae are shown with purple and green background, respectively. The motif in plant IP3K proteins were identified by MEME program. Different motif numbered 1–6 has different colors

An analysis of gene structure in plant IP3Ks was conducted (Fig. 3A, Table S1). In lower plants (Chlorophyta, Bryophyta, Pteridophyta, Gymnosperm), high number of exons were identified in *IP3Ks*, ranging from 6 to 9, except *O.lucimarinus IP3K* (*OlIPMK*) which contains only one exon. In angiosperms, most *IP3Ks* (88/97) have only one exon. Therefore, the gene structure of *IP3Ks* are different between lower and higher plants, with angiosperm *IP3Ks* display simple gene structures. To investigate the protein sequence features of IP3Ks, 6 motifs were predicted by the MEME tool (Fig. 3B). Majority of IP3Ks (92/104) contained all six motifs, while the other members contained variable numbers of motifs, such as motif1 and motif2 were lost in maize IP3Ks, motif5 was lost in AcIP3K-C (AcIPK2C), PvIP3K (PvIPK2), GmIP3K (GmIPK2), and motif6 was lost in BraIP3K2β2 (Fig. 3B). In addition, lower plants display a higher frequency of motif lost than that in higher plants. Therefore, significant differences in gene structure and motif composition were identified between lower plants and higher plants, suggesting diversity in protein function.

### Expansion of IP3Ks during plant evolution

**Fig. 4:**
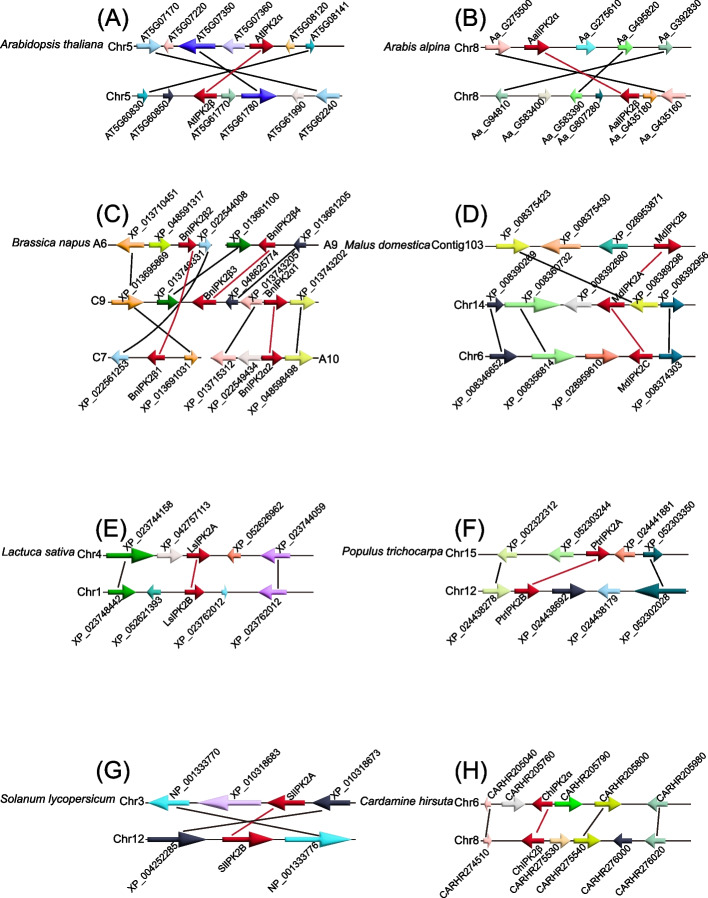
Intraspecies syntenic relationships of *IP3K* genes in representative plants. At, *A. thaliana*; Aa, *A. alpina*; Bn, *B. napus*; Md, *M. domestica*; Ls, *L. sativa*; Pt, *P. trichocarpa*; Sl, *S. lycopersicum*; Ch, *C. hirsuta.* The synthenic paralog of *IP3K* genes are connected by red lines

Tandem and segmental duplications played important roles in gene amplification [69]. *IP3K* genes were amplified in Brassicaceae. To explore the expansion of *IP3K* genes in Brassicaceae, we conducted a synteny analysis (Fig. 4, Fig. S1, Table S2). The results showed that *IP3K* isoforms were located in the syntenic blocks, and 1, 1, 3, 2, 1, 1, 1, and 1 pairs of segmental duplication genes were identified in *A. thaliana*, *A. alpina*, *B. napus*, *M. domestica*, *L. sativa*, *P. trichocarpa*, *S. lycopersicum*, and *C. hirsuta*, respectively (Fig. 4, Fig. S1, Table S2). These results suggest that in Brassicaceae, segmental duplication is the major mode of *IP3K* gene expansion.

We analyzed the Ka/Ks ratios (non-synonymous substitution rate/synonymous substitution rate) to study the selection pressure on gene evolution (Table S3) [70]. A Ka/Ks ratio greater than 1 indicates positive selection, while a ratio less than 1 suggests purifying or negative selection [70]. Our findings showed that all *IP3K* paralogs underwent purifying selection during evolution, as their Ka/Ks ratios were less than 1. The point of divergence of the duplicated *IP3Ks* was calculated based on the Ks value. In most species, the average divergence time of *IP3K* paralogous occurred approximately 30 million years ago (MYA). In *B.napus* and *M. domestica*, the divergence times of *IP3K* paralogous were later, occurring approximately 5 million years ago.

**Fig. 5:**
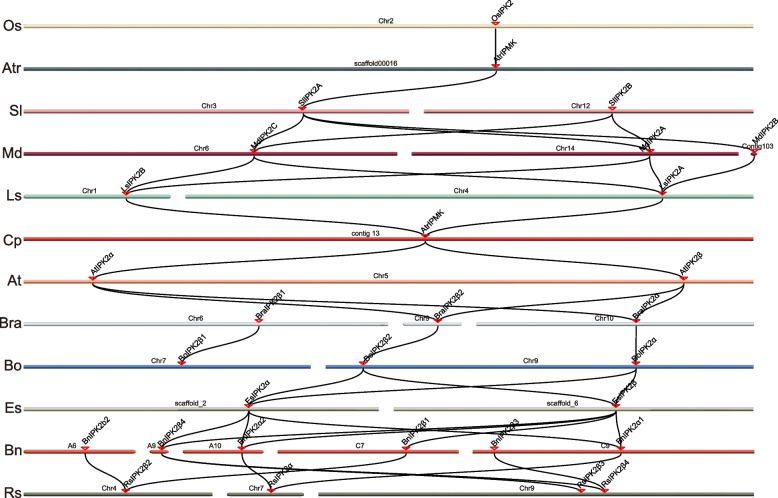
Interspecies syntenic relationships of *IP3K* genes in plants. *IP3Ks* are anchored based on their positions on the chromosomes. Black lines highlights syntenic IP3K pairs. Os: *O. sativa*; Atr: *A. trichopoda;* Sl: *S. lycopersicum*; Md:* M. domestica*; Ls:* L. sativa*; Cp: *C. papaya*; At: *A. thaliana*; Bra: *B. rapa*; Bo: *B. oleracea*; Es: *E. salsugineum*; Bn: *B. napus*; Rs: *R. sativus*

To further understand the putative clues of evolutionary events, we performed multicollinearity analyses of *IP3K* orghologous from 12 angiosperm species (Fig. 5, Table S4). Individual *IP3K* homologous genes showed one-to-one collinear relationships between *A. trichopoda* and *S. lycopersicum*, *O. sativa* and *A. trichopoda*, *B. rapa* and *B. oleracea* (Fig. 5). In addition, either one-to-many or many-to-one homozygosity was identified between *S. lycopersicum* and *M. domestica*, *M. domestica* and* L. sativa*, *A. thaliana* and *B. rapa*, *E. salsugineum* and *B. napus*, *B. napus* and *R. sativus* (Fig. 5, Table S4). A high collinearity was identified among Brassicaceae species. These results further suggested that segmental duplication contributed predominantly to expansion of *IP3K* genes.

### Phylogenetic classification of the animal IP3Ks

**Fig. 6:**
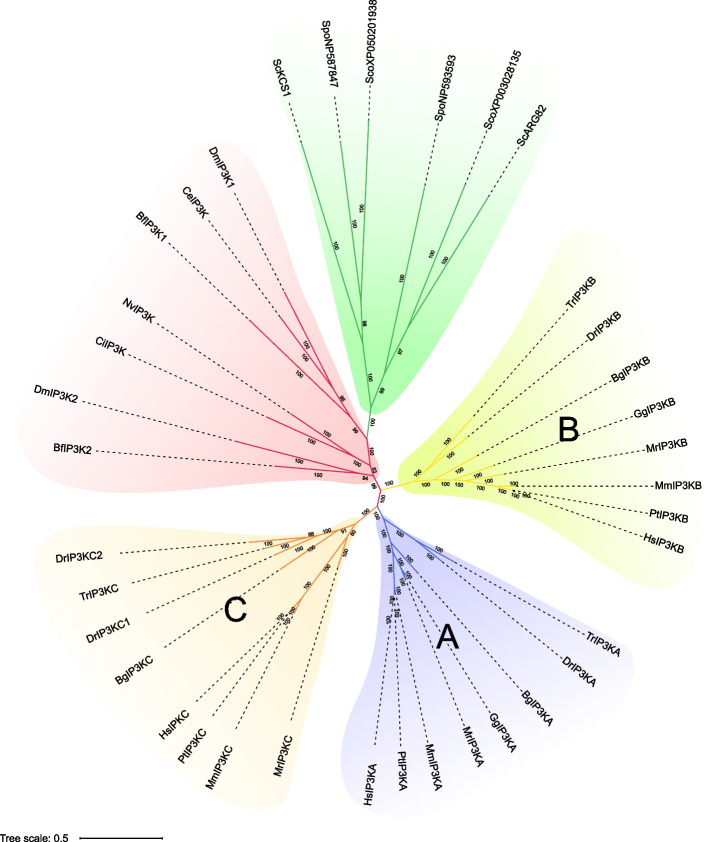
Phylogenetic relationship of IP3Ks in animals and fungi. Bayesian tree construction was performed utilizing the protein sequences of IP3K. The IP3Ks derived from different lineages are shown in different colors. Ce: *C. elegans*, Nv: *N. vectensis*, Ci: *C. intestinalis*, Dm: *D. melanogaster*, Bf: *B. floridae*, Hs: *H. sapiens*, Pt: *P. troglodytes*, Mm: *M. musculus*, Dr: *D. rerio*, Bg: *B. gargarizans*, Tr: *T. rubripes*, Mr: *M. reevesii*, Gg: *G. gallus*, Sc: *S. cerevisiae*, Sco: *S. commune*, Spo: *S. pombe*

To better understand the evolutionary relationships of animal *IP3Ks*, we construct a Bayesian tree with IP3K sequences from 13 animals and 3 fungi (also known as IPMK in fungi) (Fig. 6). Animal and fungal IP3Ks display different evolutionary patterns (Fig. 6). In invertebrates, only one or two copies of *IP3K* were identified, such as a single copy of *IP3K* existed in *C. elegans*, *N. vectensis*, *C. intestinalis*, and two copies of *IP3K* existed in *D. melanogaster* and *B. floridae* (Fig. 6). In vertebrate, IP3K was amplified and displayed three groups named IP3K-A, IP3K-B, and IP3K-C. IP3K-A forms a sister group to IP3K-C, with IP3K-C being sister to this combined group, probably due to the earlier of the two whole genome duplications (WGDs) in early vertebrates (Fig. 6) [71]. These results suggest that the diversification of vertebrate *IP3K* occurs before the formation of vertebrate species and after the formation of invertebrate species.

**Fig. 7:**
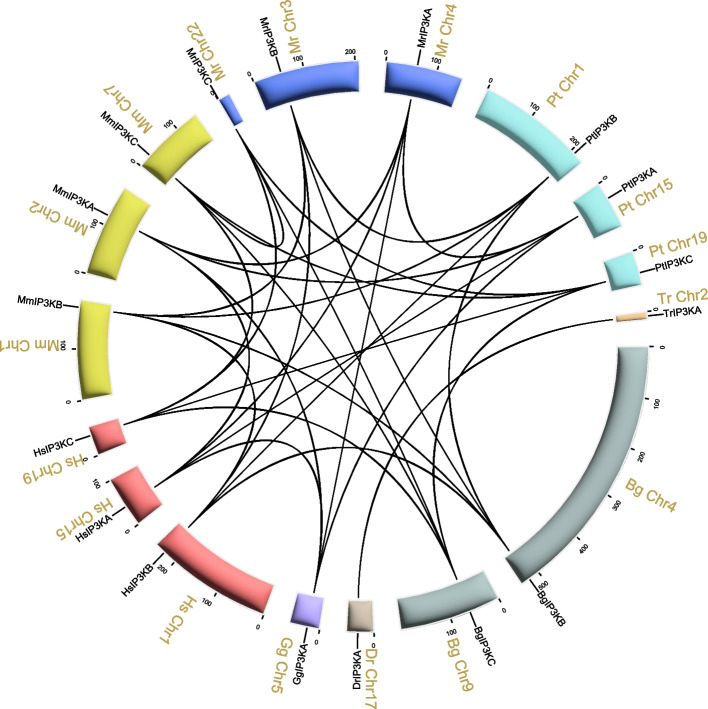
Interspecies syntenic relationship of *IP3K* genes in animals. Black lines highlights syntenic IP3K pairs. Hs: *H. sapiens*; Pt: *P. troglodytes*; Mm: *M. musculus*; Mr: *M. reevesii*; Bg: *B. gargarizans*

To further investigate the evolutionary relationships of *IP3Ks* within the vertebrate species, we performed a synteny analysis (Fig. 7, Table S4). The results showed that individual *IP3K* homologous gens showed one-to-one homozygosity, with *IP3KA* exhibits interspecies synteny exclusively with *IP3KA*, *IP3KB* demonstrates interspecies synteny exclusively with *IP3KB*, and *IP3KC* displays interspecies synteny specifically with *IP3KC* (Fig. 7). For example, in humans and mice, three pairs of *IP3K* orthologous gene pairs were observed (*HsIP3KA/MmIP3KA*, *HsIP3KB/MmIP3KB*, *HsIP3KC/MmIP3KC*). The presence of synteny connections among vertebrates suggests that whole-genome duplication contributes, in part, to the expansion of the *IP3K*.

### Gene structure and conserved motif analysis of animal IP3Ks

**Fig. 8:**
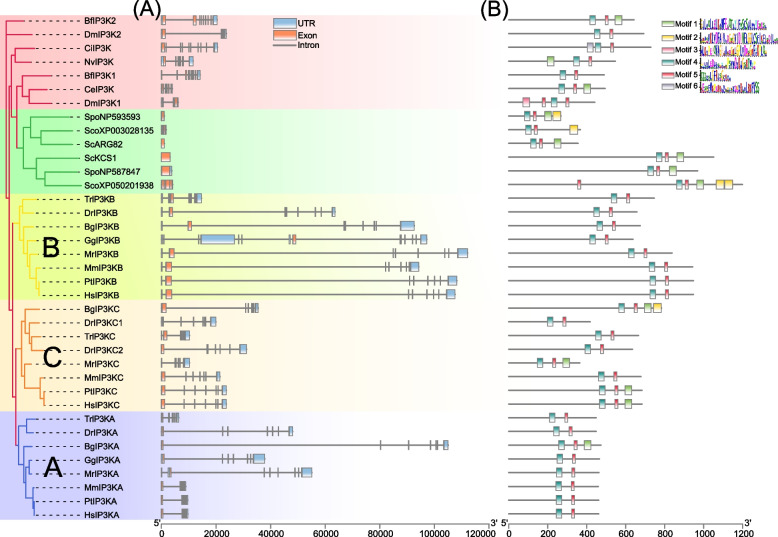
**A** Exon/intron structure analysis of animals and fungi IP3K genes. **B** Conserved motifs of animals and fungi IP3K proteins. Different colors and numbers represent different motifs. The green square background indicates the fungal category. The red square background indicates the invertebrate category. Yellow, orange and blue square backgrounds indicate vertebrate categories

To uncover the structural traits of animal IP3Ks, we constructed their intron–exon arrangements (Fig. 8A). Significant differences in the number of exons and gene lengths were identified in animal and fungal *IP3Ks*. The fungal *IP3Ks* displayed short gene length and had no intron, while most animal *IP3Ks* (23/31) displayed similar exon/intron structures containing six introns. In vertebrates, the *IP3KB* clade displayed longer gene length than *IP3KA* and *IP3KC* clades. In conclusion, animal and fungal *IP3Ks* showed different exon/intron structures and gene length, suggesting the diversity of *IP3K* genes the evolution.

The conserved motifs were predicted by MEME as plant IP3Ks (Fig. 8B). Most of the animal and fungal IP3Ks exhibited similarities in motif composition. Motif4 and motif5 are found to be the common among all proteins, indicating their highly conserved domain. Fungal IP3Ks showed a more diversity in motif composition, with ScoXP003028135 harbored two repeated motif5 and motif2, respectively. Compared with plant IP3Ks, many motifs were lost in animal and fungal IP3Ks, implying the functional diversity of *IP3K* gene family among fungal, animal and plant.

### Gene expression profiles of IP3Ks in evolutionarily important lineages of green plants and animals

**Fig. 9:**
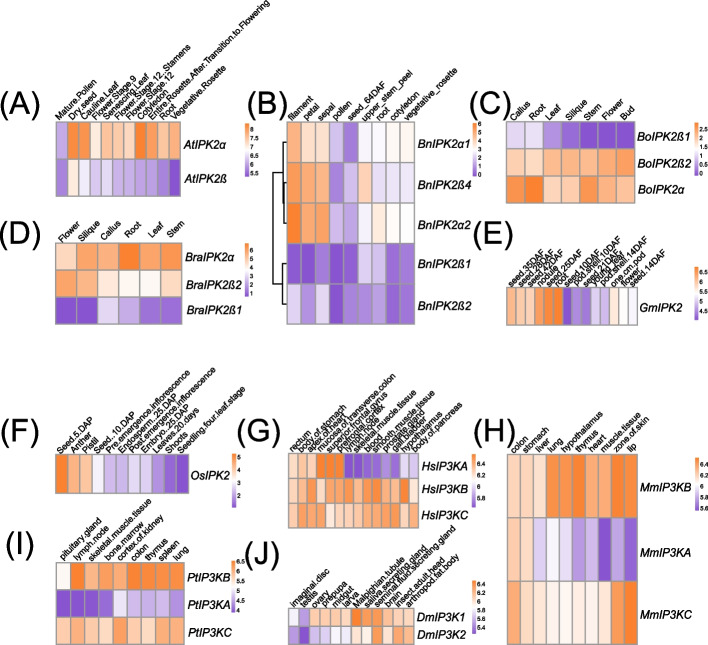
Expression of *IP3Ks* in plants and animals. **A**, *A. thaliana*; **B**, *B. napus*; **C**, *B. oleracea*; **D**, *B. rapa*; **E**, *G. max*; **F**, *O. sativa*; **G**, *H. sapiens*; **H**, *M. musculus*; **I**, *P. troglodytes*; **J**, *D. melanogaster*

To explore the expression of *IP3K* genes, we conducted gene expression analysis of *IP3Ks* in ten representative plant and animal species (*A. thaliana*, *B. napus*, *B. oleracea*, *B. rapa*, *G. max*, *O. sativa*, *H. sapiens*, *M. musculus*, *P. troglodytes*, and *D. melanogaster*) (Fig. 9, Table S5). In *A. thaliana*, 11 tissues and developmental stages were investigated (Fig. 9A). *AtIPK2α* and *AtIPK2β* are expressed in a variety of tissues, and *AtIPK2α* is expressed high than *AtIPK2β* in dried seeds, stamens, cotyledons, roots, and mature pollen. In dry seeds and cauline leaves, *AtIPK2β* expression was high than *AtIPK2α*. These results suggesting that this paralogous gene pairs have functional divergence, consistent with AtIPK2α functions in pollen germination and root growth [51], and AtIPK2β functions in branching, flowering, and seedling development [52–54]. *B. napus* contained the largest number of *IP3K*, but most of them have low expression levels among the nine tissues examined (Fig. 9B). In filaments, petals, and sepals, *BnIP3K2α2*, *BnIP3K2ß4*, and *BnIP3K2α1* showed significantly high specific expression than other genes. In addition, in *B. oleracea* and *B. rapa*, *IP3K2α* and *IP3K2β* genes were widely expressed in all tissues, whereas the expression level of *IP3K2α* was significantly higher than that of *IP3K2β* (Fig. 9C-D). In summary, in Brassicaceae, the duplicated *IP3K* genes differ in expression in different tissues, suggesting functional divergence between these paralogs. In *G. max*, the *IP3K* (*GmIPK2*) gene showed specific high expression in seeds, nodules, and roots; whereas in *O. sativa*, the *IP3K* (*OsIPK2*) gene showed specific high expression in seeds, anthers, and pistils (Fig. 9E-F), suggesting that these *IP3K* genes have a potential role in organ development.

*IP3K* undergoes two expansions in vertebrates, yielding three copies (*IP3KA/B/C*). In *H. sapiens*, *M. musculus*, and *P. troglodytes*, *IP3KB* and *IP3KC* are widely expressed in almost all tissues, with *IP3KB* displayed high expression level than *IP3KC* (Fig. 9G-I). *IP3KA* showed obvious tissue specificity, for example, *HsIPKA* was specifically expressed in the superior frontal gyrus, mucosa of the transverse colon, and the prefrontal cortex. These expression pattern consistent with its functional divergence. A similar expression divergence was also identified in *D. melanogaster IP3Ks* (Fig. 9J). The *DmIP3K1* and *DmIP3K2* genes showed widespread expression in several tissues, with *DmIP3K1* expression levels significantly higher than *DmIP3K2*. *DmIP3K2* is specifically highly expressed in the seminal fluid secreting gland, insect adult head, and arthropod fat body. In summary, both plant and animal *IP3K* genes experienced functional divergence after duplication.

### Sequence analysis for functional diversification of IP3Ks

**Fig. 10:**
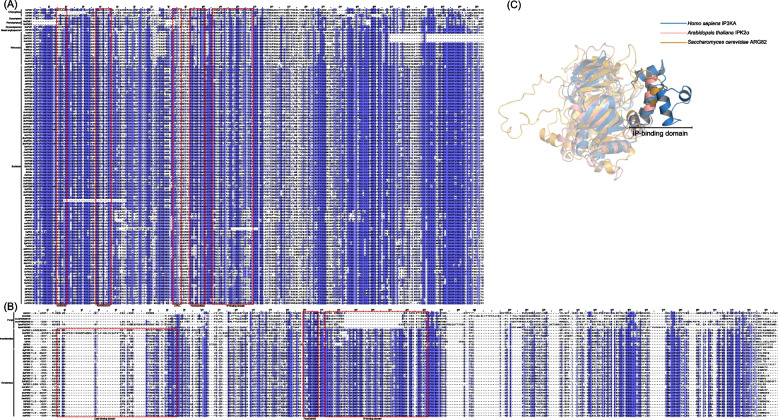
**A**, **B** Multiple sequence alignment and conserved motifs in IP3K proteins. Conservative motifs are boxed out using red dashed lines. **C** *A. thaliana*, human, and yeast IP3K protein tertiary structure overlay diagrams were constructed using pymol. The opaque portion indicates the IP-banding domain

Multiple sequence alignment was performed using the M-Coffee web server for IP3K proteins (Fig. 10A) [72, 73]. A common motif, PxxxDxKxG, which serves as a signature for binding inositol phosphates, was existed in all IP3K proteins [17, 25]. Apart from SiIPK2α, all plant IP3K sequences contained a core catalytic tyrosine kinase motif RxxxExxxY, suggesting that they are tyrosine-specific protein kinases [74]. The IP3K sequences from Solanaceae and Rosaceae families possess a Glycine-rich consensus ATP-binding GxGxxG motif, which is a characteristic feature of the protein kinase C (PKC) catalytic domain, indicating that these IP3Ks can be phosphorylated by PKC [75]. In angiosperms, most of the IP3K sequences have protein-recognizing LxxLL motifs, suggesting that they are involved in participating in protein–protein interactions [76]. Interestingly, these conserved motifs have not been detected in fungi and animal IP3Ks (Fig. 10A and B).

In addition, the IP-binding domains are highly conserved in plant and fungal IP3K, which differs from animal IP3Ks (Fig. 10A-C) [23, 60, 61]. Animal IP3K contains a large inserts in the IP-binding domain, suggesting a broader substrate selectivity than plant and fungal IP3Ks (Fig. 10A-C). A conserved Ca^2+^/Calmodulin (CAM) binding domain was identified in the N-terminal of animal IP3Ks, while *A. thaliana*, yeast, and nematode IP3K lack this domain (Fig. 10B). These differences suggest that animal IP3Ks are activated by CaM in a Ca^2+^-dependent manner, while *A. thaliana*, yeast, and nematode IP3Ks remain insensitive to Ca^2+^/CaM.

## Discussion

In this study, we performed a comprehensive evolutionary analysis of IP3Ks in green plants and animals. The phylogenetic insights provide valuable information for future molecular and biological studies of various IP3K proteins.

### Phylogenetic relationship of IP3Ks

**Fig. 11:**
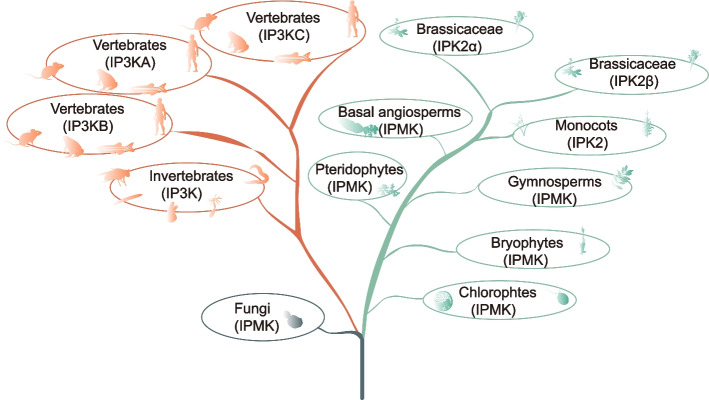
A proposed evolutionary model of IP3Ks in animals, plants, and fungi. IP3Ks in plants and fungi are also known as IPMKs. The model is based on the phylogeny of IP3Ks and the cladogram of animals and plants. The origin of IP3Ks can be traced back to before the divergence of plant and animal fungal species

*IP3K* genes are widely distributed among fungi, plant and animal lineages. The IP binding domain with a consensus sequence PxxxDxKxG is highly conserved in fungi, animals and plants [25], indicating that it originate from a common ancestor before the divergence of fungi, animals and plants, which is consistent with former results [10]. The *IP3K* gene maintained low-copy numbers in fungi, animals and plants, suggesting for functional conservation during evolution. Yeast, *A. thaliana*, human and rat IP3Ks were reported to function in phosphatidylinositol signaling by phosphorylating a same substrate-inositol 1,4,5-trisphosphate (IP_3_) [23, 77, 78], suggesting that IP3K-mediated phosphatidylinositol signaling is conserved and essential for growth and development. Previous studies have shown that the IPK family shares an evolutionary ancestry and that IP3Ks are the most recent evolutionary branch of the IPK family, as they are restricted to metazoans [23, 77, 79]. Based on the phylogenetic analysis, we proposed a model for the evolution of *IP3K* genes in plant and animal lineages (Fig. 11). Our analysis supports an ancestry origin of *IP3K* genes that the *IP3K* gene origin can be traced back to the common ancestor before the divergence of fungi, plants and animals. IP3K genes expanded during the histories of higher plants and vertebrates, respectively. In Brassicaceae, *IP3K* underwent one duplication forming the *IPK2α* and *IPK2β* branches, while in vertebrates, *IP3K* underwent two expansions forming three clads (Fig. 11). The synteny analysis indicated that these duplications were derived from large-scale duplication events such as whole genome duplications (WGDs) or segmental duplications. It has previously been reported that regulatory genes and signalling genes are more likely to be retained after duplication events compared to the genome-wide average [80, 81]. The fact that *IP3K* genes function mainly in phosphatidylinositol signalling regulation is another excellent example.

### Functional diversification of IP3Ks

The long evolutionary history of *IP3K* genes allowed a great differences in gene structures and sequence features, resulting in differences in expression patterns and diversification in physiological functions. The IP-binding domain of animal IP3Ks is larger compared to that of fungal and plant IP3Ks, allowing for a wider selection of substrates [60, 61]. Plant and animal IP3Ks displayed significant differences in domain structure, such as plant IP3Ks contain a conserved core tyrosine kinase catalytic motif (RxxxExxxY) and a protein-recognition motif (LxLL), Solanaceae and Rosaceae IP3K contained a conserved GxGxxG motif for protein kinase C (PKC) recognition [72, 75], and mammalian IP3K contained Ca^2+^/calmodulin (CAM) binding domain [65, 82]. Compared to plant IP3Ks, many motifs were lost in animal and fungi IP3Ks (Figs. 3 and 8), such as animal IP3K generally does not contain motif1, motif2, motif3, and motif6 (Fig. 8). In addition, fungi, animal and plant *IP3Ks* showed significant differences in gene structures, with most fungi and angiosperm *IP3Ks* have no introns, while animal and lower plants *IP3Ks* contain more introns (Figs. 3 and 8). These results suggest that *IP3K* structures differ significantly among plants, animals, and fungi, which may reflect the need for them to carry out different functions in different organisms.

Although fungi, animal and plant IP3Ks can phosphorylate IP_3_, their catalytic activity and substrates were obviously different. Yeast and plant IP3Ks displayed multikinase activity with a broad inositol phosphates [24]. Both yeast and *A. thaliana* IP3Ks displayed a dual-specificity IP_3_/IP_4_ 6/3-kinase activity that sequentially phosphorylates IP_3_ to 1,4,5,6-tetrakisphosphate (IP_4_) to 1,3,4,5,6-pentakisphosphate (IP_5_) [17, 25, 29]. However, human and rat IP3Ks specifically phosphorylates IP_3_ at the 3-OH group to yield 1,3,4,5-tetrakisphosphate (IP_4_) [83–85]. Therefore, animal, plant, and fungi IP3Ks display divergence in biochemical activity, which further reflect the functional divergence in physiology. However, the in vivo catalytic activities of IP3Ks were only reported in part species, especially yeast, rat, human and *A. thaliana*. It is important to characterize and analysis new IP3Ks from more species, such as algae, moss, and invertebrates. In addition, specific investigation of the relationship between the kinase activities and biological functions of IP3Ks is required to demonstrate in more species.

*IP3Ks* are involved in a wide range of biological processes. Expression analyses revealed several instances of tissue-specific expression, revealed functional specificity of different *IP3K* isoforms (Fig. 9). For instance, *A. thaliana AtIPK2α* had relatively high transcript levels in pollen grains, flowers, roots, and leaves, while *AtIPK2β* was weakly expressed in pollen grains and flowers (Fig. 9A). These expression patterns were consistent with previous functional studies. Inhibit of *AtIPK2α* promoted pollen grain germination and pollen tube growth [51], while knockout *AtIPK2β* promoted flowering, enhanced sensitivity to glucose and decreasing branching [52–54]. In addition, in *atipk2α atipk2β* double mutant, pollen development and pollen tube guidance were impaired [86]. These results showed that *AtIPK2α* and *AtIPK2β* have both redundant and divergent roles. Compared with *A. thaliana IP3K*s, the three human and rat P3K isoforms (A, B, and C) displayed significant functional divergence [33–39]. For instance, *HsIP3KB* was expressed at significantly higher levels than *HsIP3KA* and *HsIP3KC* in all tested tissues (Fig. 9G). HsIP3KB has a more complex subcellular localization, localized in the plasma membrane, cytoskeleton, and endoplasmic reticulum, with HsIP3KA being associated with the cytoskeleton, whereas HsIP3KC is exclusively present in the cytoplasm [87]. These difference suggest different functions of these three isoforms. *HsIP3KB* plays an important role in the development of immune cells [88], while *HsIP3KA* stimulates tumor cell migration [43, 89]. All of these results indicate that there are significant differences in the structures of plant and animal *IP3Ks*, and *IP3Ks* are functionally differentiated within their respective species.

## Conclusion

Our analysis advanced the knowledge and concept of IP3K evolution. IP3Ks are ubiquitious in all eukaryotic species examined to date, from yeast to plants to humans. We have revealed marked differences in gene structures among yeast, plant, and human IP3Ks, which advanced our understanding of the molecular details governing their different catalytic activities and biological functions. Current knowledge of IP3Ks has been derived mainly from yeast, rat, human and *A. thaliana*. More additional researches are required to identify the new IP3Ks from a wider range of species and demonstrate their novel kinase activities in vivo. Enhanced knowledge of the evolution, structure and function of IP3K will facilitate targeted pharmacological and agronomical interventions to modulate these crucial IP3K activities.

## Materials and methods

### Data sources and sequence acquisition

We selected a total of 73 species and acquired their genome and proteome sequences from public databases such as NCBI (http://www.ncbi.nlm.nih.gov/), Ensembl Plants (http://plants.ensembl.org/index.html), and BRAD (http://brassicadb.cn/#/) (Table S6). Two methods are available to identify IPK genes in animals, plants, and fungi. To identify the IPK domain, we initially conducted the HMMER search (E-value = 1e-10) using the Hidden Markov Model profile of the IPK domain (PF03770) in local databases. Additionally, we employed the Basic Local Alignment Search Tool algorithms (BLASTP) with the amino acid sequences of Escherichia coli MscS and *A. thaliana* IPK members against the protein database, setting an E-value threshold of less than 1e-6. The putative IPKs were further validated with online tools CDD (https://www.ncbi.nlm.nih.gov/Structure/cdd/wrpsb.cgi/.) [90], HMM (https://hmmer.org/) [91] and SMART (https://smart.embl-heidelberg.de/) [92].

### Multiple sequence alignment, protein structure predictions, and phylogenetic analysis

IP3K multiple sequence alignments were performed using MAFFT software [93]. Phylogenetic trees were generated based on the IPK full protein sequences. The maximum likelihood (ML) phylogenetic tree was constructed using IQ-TREE with the parameter '-m MFP -bb/alrt 1000' and 1000 ultra-bootstrap replicates [94]. Bayesian trees were constructed using MrBayes 3.2.1 using a mixed model until the mean standard deviation of split frequencies < 0.01 [95]. SWISS-MODEL was used to model the homology of protein structure [96]. The crystal structure was visualized using PyMol [97].

### Synteny and Ks analysis

To identify homologous pairs across different species and within a specific species, we utilized the all-to-all BLASTP method. Syntenic blocks were then inferred using MCScanX with default parameters, including an E-value threshold of 1e-10 and a minimum of 5 BLAST hits [98]. The resulting synteny map was visualized using CIRCOS, where putative duplicated genes were connected by lines to illustrate their relationships [99].

The biological significance of Ks can be utilized to estimate the divergence time of significant genome-wide and segmental duplication events within a species during the process of evolution, which can then be calculated [100]. The divergence time was determined using the formula *T* = *Ks/2r*, where Ks represents the synonymous substitutions per site and r represents the rate of divergence for nuclear genes in plants. The value of r, assumed to be the synonymous substitutions per site per year, was 1.5 × 10^–8^ for dicots [101], 6.5 × 10^–9^ for Poaceae [102], and 4.79 × 10^–9^ for ferns [103].

### Expression analysis of IP3K genes

Expression data for ten species were downloaded from public databases including *A. thaliana* (The Arabidopsis Information Resource, https://www.arabidopsis.org/), *B. napus* [104], *B. oleracea* [105], *B. rapa *[106], *G. max *[107], *O. sativa* [108], *H. sapiens*, *M. musculus*, *P. troglodytes*, and *D. melanogaster* (Table S5) [109]. A heatmap, generated using R software with k-means clustering, was created.

### Supplementary Information

**Supplementary Material 1.**

**Supplementary Material 2.**

## Data Availability

The datasets used and/or analyzed during the current study available from the corresponding author on reasonable request. All raw sequencing data were downloaded from public database. The detailed information could be found in Supplementary Table S6. IP3K protein sequences are available in Zenodo (https://zenodo.org/records/10589255).
