# Supplementary material for: Origin, evolution, and diversification of inositol 1,4,5-trisphosphate 3-kinases in plants and animals
Source: BMC Genomics. 2024 Apr 8;25:350. doi: 10.1186/s12864-024-10257-7 (PMC11000326; doi:10.1186/s12864-024-10257-7)
Supplement: Supplementary file 1 — Supplementary Material 1. [file 12864_2024_10257_MOESM1_ESM.pdf]

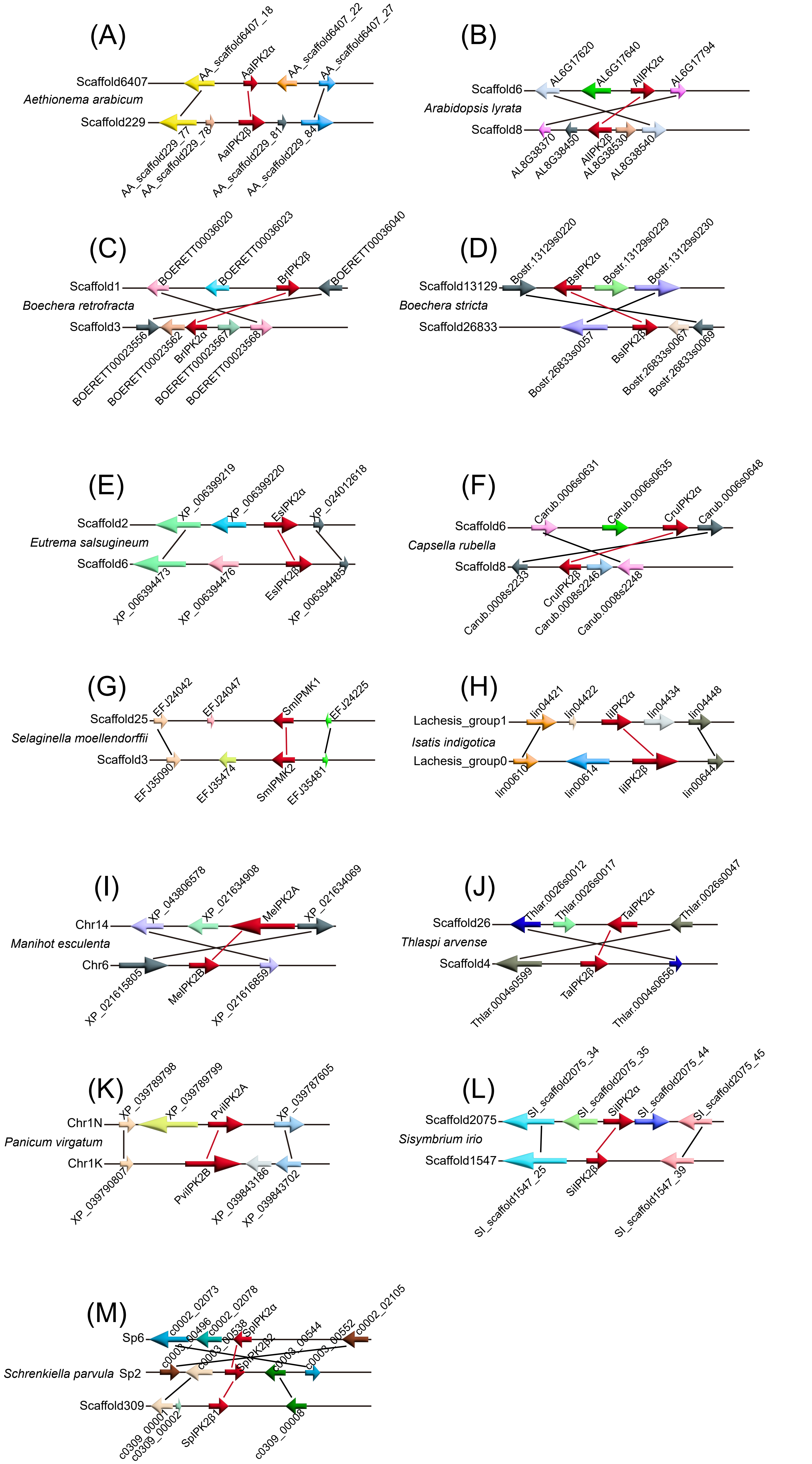

Figure S1. Intraspecies syntenic relationships of *IP3K* genes in various plant species. (A), *Aethionema arabicum*; (B), *Arabidopsis lyrata*; (C), *Boechera retrofracta*; (D), *Boechera stricta*; (E), *Eutrema salsugineum*; (F), *Capsella rubella*; (G), *Selaginella moellendorffii*; (H), *Isatis indigotica*; (I), *Manihot esculenta*; (J), *Thlaspi arvense*; (K), *Panicum virgatum*; (L), *Sisymbrium irio*; (M), *Schrenkiella parvula*.
